# Visible/near-infrared hyperspectral imaging combined with machine learning for identification of ten *Dalbergia* species

**DOI:** 10.3389/fpls.2024.1413215

**Published:** 2024-05-31

**Authors:** Zhenan Chen, Xiaoming Xue, Haoqi Wu, Handong Gao, Guangyu Wang, Geyi Ni, Tianyi Cao

**Affiliations:** ^1^ College of Criminal Science and Technology, Nanjing Police University, Nanjing, China; ^2^ College of Forestry and Herbgenomics, Nanjing Forestry University, Southern Tree Seed Inspection Center, National Forestry and Grassland Administration, Co-Innovation Center for Sustainable Forestry in Southern China, Nanjing, China; ^3^ Faculty of Forestry, The University of British Columbia, Vancouver, BC, Canada; ^4^ Key Laboratory of Wildlife Evidence Technology State Forest and Grassland Administration, Nanjing Police University, Nanjing, China; ^5^ College of Landscape and Architecture, Nanjing Forestry University, Nanjing, China

**Keywords:** PLS-DA, SVM, CNN, wood identification, rosewood

## Abstract

**Introduction:**

This study addresses the urgent need for non-destructive identification of commercially valuable *Dalbergia* species, which are threatened by illegal logging. Effective identification methods are crucial for ecological conservation, biodiversity preservation, and the regulation of the timber trade.

**Methods:**

We integrate Visible/Near-Infrared (Vis/NIR) Hyperspectral Imaging (HSI) with advanced machine learning techniques to enhance the precision and efficiency of wood species identification. Our methodology employs various modeling approaches, including Principal Component Analysis (PCA), Partial Least Squares Discriminant Analysis (PLS-DA), Support Vector Machine (SVM), and Convolutional Neural Networks (CNN). These models analyze spectral data across Vis (383–982 nm), NIR (982–2386 nm), and full spectral ranges (383 nm to 2386 nm). We also assess the impact of preprocessing techniques such as Standard Normal Variate (SNV), Savitzky-Golay (SG) smoothing, normalization, and Multiplicative Scatter Correction (MSC) on model performance.

**Results:**

With optimal preprocessing, both SVM and CNN models achieve 100% accuracy across NIR and full spectral ranges. The selection of an appropriate wavelength range is critical; utilizing the full spectrum captures a broader array of the wood's chemical and physical properties, significantly enhancing model accuracy and predictive power.

**Discussion:**

These findings underscore the effectiveness of Vis/NIR HSI in wood species identification. They also highlight the importance of precise wavelength selection and preprocessing techniques to maximize both accuracy and cost-efficiency. This research contributes substantially to ecological conservation and the regulation of the timber trade by providing a reliable, non-destructive method for identifying threatened wood species.

## 1 Introduction

Rosewood ranks among the most trafficked commodities, both in terms of value and volume, comparable to notorious items such as rhinoceros horn, elephant ivory, and tiger fur (Soudier et al., 2022). Its distinctive texture and color have made it a favored material for artwork and furniture (Barrett et al., 2013; McClure et al., 2015). The *Dalbergia* genus, encompassing various rosewood species, thrives in subtropical and tropical areas and is a prominent part of the timber trade. Certain species of *Dalbergia* are particularly prized for their valuable heartwood and medicinal qualities (Son et al., 2017). However, rampant over-exploitation has put the *Dalbergia* genus at risk of extinction, prompting its inclusion in the CITES (Convention on International Trade in Endangered Species of Wild Fauna and Flora) list.

Despite their economic and ecological importance, the identification of *Dalbergia* species remains challenging due to their similar visual and anatomical features. Traditional wood identification methods such as microscopy can classify at the genus level but often lack the precision and efficiency required for accurate species differentiation (Ravindran et al., 2020). These methods are generally destructive, necessitating extensive sample preparation and lengthy analysis times, which is not sustainable (Gasson et al., 2010).

In contrast, Visible/Near-Infrared (Vis/NIR) Hyperspectral Imaging (HSI) technology offers a non-destructive, efficient, and environmentally friendly alternative (**Table 1**). This technology is complemented by advanced machine learning techniques that include Principal Component Analysis (PCA), Partial Least Squares Discriminant Analysis (PLS-DA), Support Vector Machines (SVM), and Convolutional Neural Networks (CNN).

**Table 1:** Comparison of traditional and spectroscopic wood identification techniques.

Attribute	Vis/NIR HSI	Traditional Wood Identification Techniques	Near-Infrared Spectroscopy
Non-destructive	Yes	No (often requires sample destruction)	Yes
Efficiency	High (rapid analysis)	Low (time-consuming processes)	High (quick data acquisition)
Environmental Impact	Low (non-invasive)	High (due to chemical usage)	Low (non-invasive)
Capability	Excellent at detecting subtle chemical variations	Limited by visual and physical properties	Good at detecting major compositional differences
Technology Requirement	Advanced (requires sophisticated equipment)	Basic (microscopes, chemical reagents)	Moderate (specialized NIR equipment required)
Data Complexity	High (complex data interpretation)	Low (simpler data)	Moderate (requires specific expertise)

These methods are regarded as sophisticated due to their robust capability to model the complex, nonlinear relationships that are typical of high-dimensional spectral data. Specifically, PCA reduces dimensionality while preserving significant variance, enabling clearer patterns in the data. PLS-DA enhances this by focusing on maximizing the separation between classes of data, crucial for accurate classification. SVM offers a powerful framework for classification and regression by constructing hyperplanes in a multidimensional space that best separates different classes. Finally, CNNs, with their deep learning structures, are particularly adept at capturing spatial hierarchies in data, which is invaluable for identifying subtle differences in hyperspectral images. Collectively, these techniques automate and refine the pattern recognition process, which is essential for distinguishing between closely related species and adapting to new data with minimal human intervention, thereby increasing the reliability and efficiency of species identification (Gold et al., 2020).

HSI technology has become indispensable in fields such as botany (Dale et al., 2013), wildlife conservation (Grabska et al., 2022), marine biology (Piarulli et al., 2022), and wood science (Ma et al., 2019). By capturing extensive chemical and spatial information across Vis to NIR spectra, HSI enables researchers to detect subtle differences in species and materials through their unique biochemical signatures. This technology is particularly effective in wood science, where it provides detailed insights into the microscopic structure and chemical alterations of wood, allowing for the identification of key constituents like lignin and cellulose which are crucial for assessing wood’s mechanical properties and durability (Wang et al., 2022; Xue et al., 2022, 2023). Despite facing challenges related to data storage and processing demands, the future of HSI in these diverse applications looks promising, thanks to ongoing advancements in sensor technology and algorithm development aimed at enhancing data handling and robustness (Amaral et al., 2021).

This study distinctly advances beyond the scope of previous research by integrating sophisticated machine learning techniques with HSI technology to target the precise identification of ten endangered *Dalbergia* species. Unlike previous studies that focused primarily on either HSI technology or basic machine learning applications, this research leverages both to achieve unprecedented accuracy and efficiency in species differentiation. This methodological innovation addresses significant gaps left by prior methodologies, particularly in the practical application of these technologies. Designed explicitly to support timber trade regulation and conservation efforts, this study translates scientific insights into actionable impacts, enhancing enforcement of trade laws and promoting sustainable forestry practices.

By focusing on a diverse range of *Dalbergia* species, our study not only extends the application of HSI in wood science but also innovatively applies machine learning to enhance species identification precision. We hypothesize that the integration of these sophisticated analytical techniques will significantly improve identification accuracy, thereby enhancing the enforcement of trade laws and promoting sustainable forestry practices.

## 2 Materials and methods

### 2.1 Samples

**Table 2** presents the scientific classification and quantity of wood samples used in this study, all of which were provided by the Nanjing Police College. Specifically, our collection includes ten distinct species of *Dalbergia*, ensuring that each species is represented by at least 18 individuals. From each individual, we obtained 5 to 9 samples, culminating in a comprehensive assortment of 800 wood samples. To ensure robust model evaluation, these samples were divided into two groups: 600 samples (75%) for the calibration set to train the models, and 200 samples (25%) for the validation set to test their performance. This division helps prevent model overfitting and guarantees effective performance on new data. The air-dried specimens, maintaining a moisture content between 11% and 11.5%, were subjected to surface smoothing through sanding to reduce surface irregularities. These samples were then prepared into pieces measuring 20 cubic mm^3^ for analysis. According to our previous research, transverse section of sapwood is more suitable for wood identification (Xue et al., 2022). In this study, spectral data was scanned in transverse section of the samples.

**Table 2:** The plant materials used in the study.

Scientific classification		Calibration Set	Validation Set
*Dalbergia tucurensis*	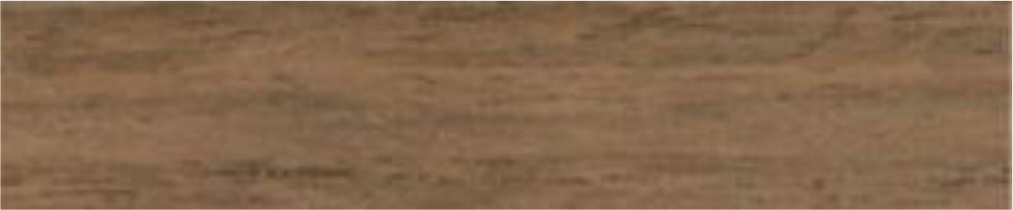	60	20
*Dalbergia cultrata*	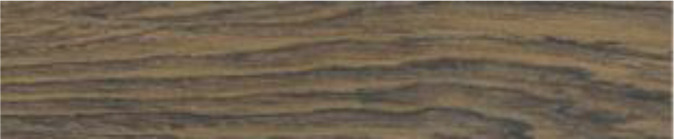	60	20
*Dalbergia latifolia*	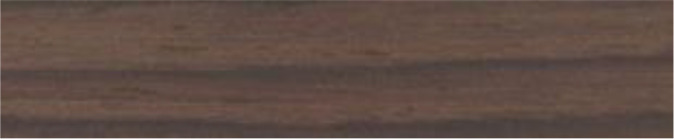	60	20
*Dalbergia stevensonii*	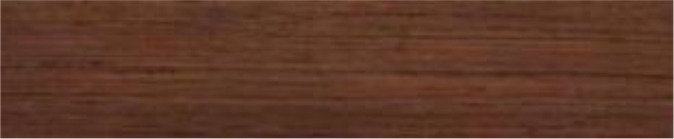	60	20
*Dalbergia bariensis*	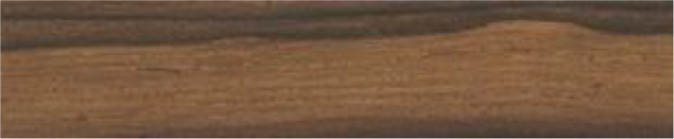	60	20
*Dalbergia cearensis*	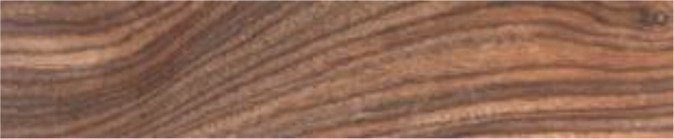	60	20
*Dalbergia cochinchinensis*	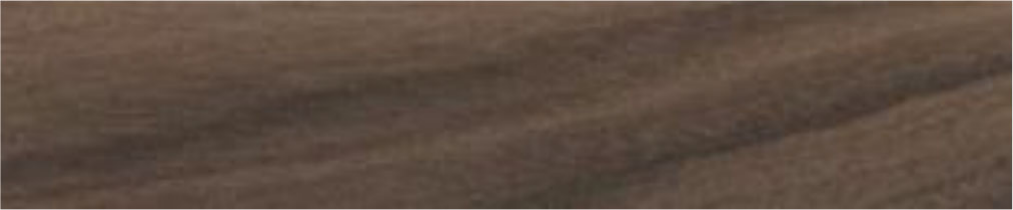	60	20
*Dalbergia oliveri*	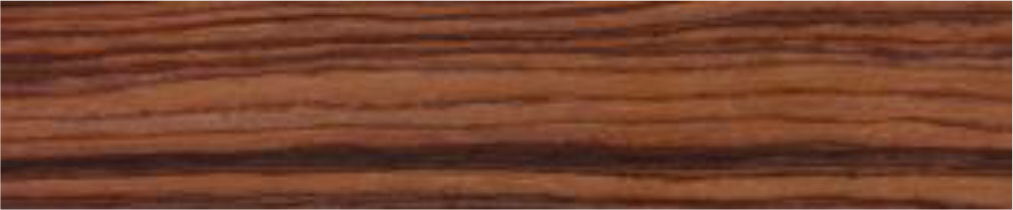	60	20
*Dalbergia retusa*	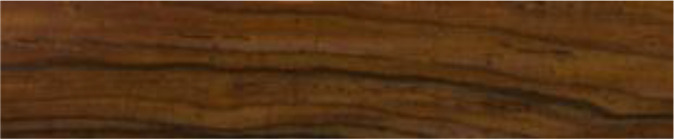	60	20
*Dalbergia congestiflora*	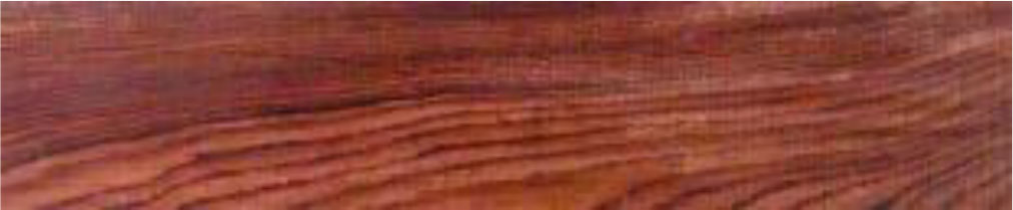	60	20

### 2.2 Equipment and spectra acquisition

**Figure 1:**
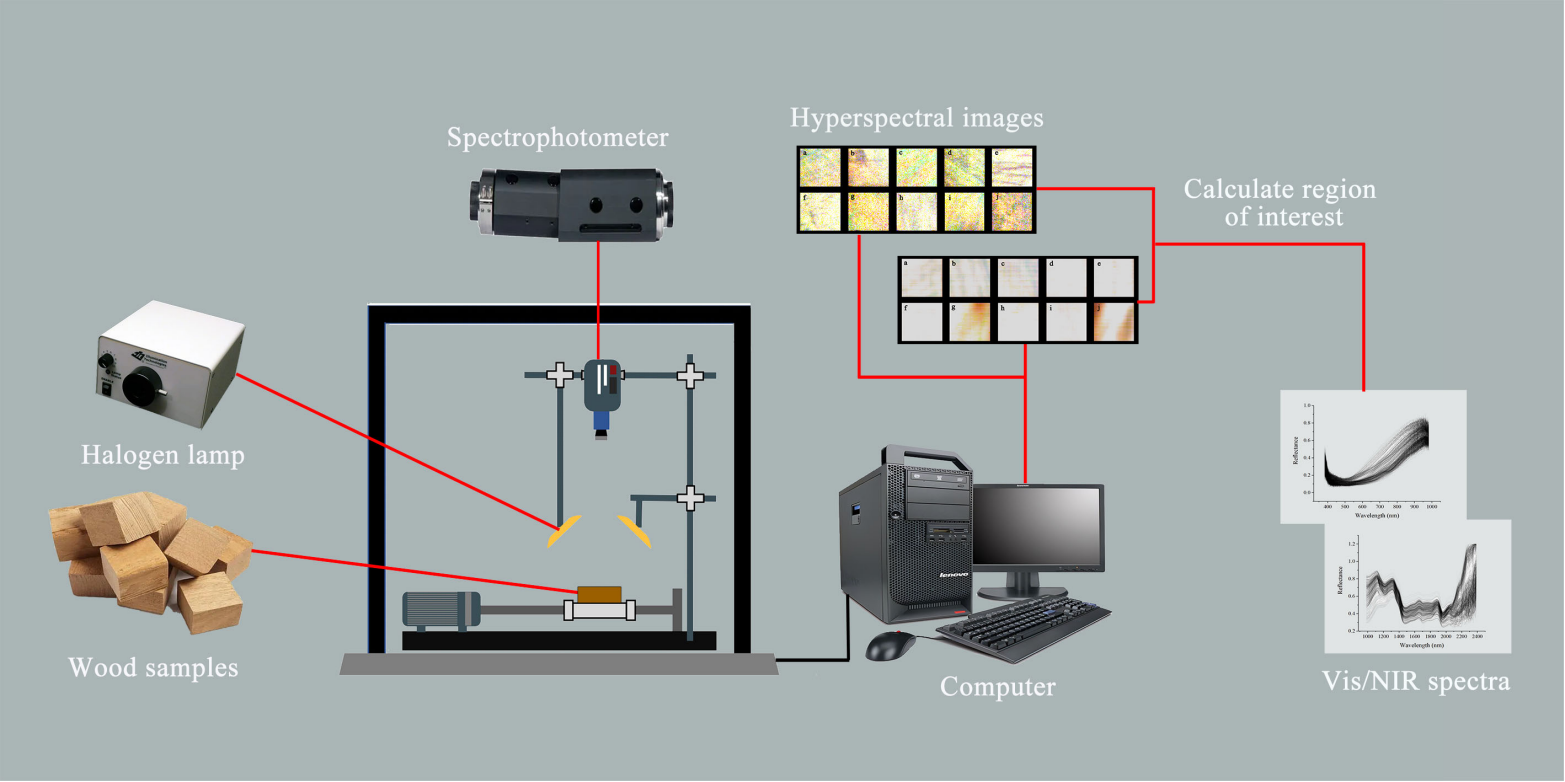
Vis/NIR-HSI system.

To collect the Vis/NIR spectra of the wood samples, the experiment was conducted in a darkroom equipped with an ImSpectorV10E Vis/NIR spectrophotometer. The samples were illuminated by a 350 W halogen lamp from Illumination Technologies, USA, positioned at a 45-degree angle, with the sample surface located 350 mm from the light source. The spectroradiometer was set approximately 170 mm from the sample’s surface for spectral analysis. The spectrometer’s detection wavelength spanned from 383 to 2386 nm. It featured a sampling resolution of 1.4 nm for the Vis spectrum and 6.2 nm for the NIR spectrum. A vital preparatory step involved preheating the halogen lamps for 15 minutes to ensure they were operating optimally for precise results. Calibration of the instrument was meticulously carried out using a black image (achieved by covering the camera lens) and a white image (using a Teflon plate with 99.9% reflectance) before the spectrum collection commenced. During the scanning and averaging process, the wood samples were positioned on a black cloth to ensure consistent conditions. The configuration of the Vis/NIR-HSI system employed in this research is illustrated in **Figure 1**.

Spectral data for the wood samples were collected across three distinct spectral ranges: Vis, NIR, and a combined Vis-NIR spectrum. The selection of these spectral ranges was strategically made to maximize the identification accuracy of *Dalbergia* species, considering their specific chemical and physical properties which are best captured at different wavelengths. The Vis spectrum (383–982 nm) primarily focuses on pigment identification, capturing the Vis light where most wood pigments absorb wavelengths, which is crucial for distinguishing subtle color variations indicative of different species (Chen et al., 2016). The NIR spectrum (982–2386 nm) is utilized for its sensitivity to molecular vibrations related to moisture content, lignin, and cellulose, essential for assessing the mechanical properties and durability of wood (Amaral et al., 2021). The combined spectrum (383–2386 nm) offers a comprehensive overview, enhancing our predictive accuracy by including both Vis and NIR advantages, allowing for the detection of a broader range of chemical markers and physical properties. This methodological approach ensures no potentially discriminative spectral features are missed, thereby enhancing the robustness and precision of our species identification process.

To ensure reliable measurements, we collected 80 spectra for each wood sample. Averaging these spectra reduces random noise and enhances the data’s consistency, critical for accurate species identification. This method increases the precision of our results by decreasing data variability, which is particularly important in distinguishing subtle differences between species. The choice of 80 spectra balances thorough data collection with efficient processing, providing a solid foundation for our analysis.

### 2.3 Model development

In this research, we developed various predictive models, including the integration of PCA with a classifier, PLS-DA, SVM, and CNN, to tackle the challenges associated with identifying wood species. PCA reduces the dimensionality of our data, retaining only essential features, thereby lowering computational demands and mitigating overfitting. The refined data are then subjected to logistic regression, noted for its efficacy in binary and multiclass problems and its capability to generate clear probability scores. PLS-DA is adept at managing high-dimensional data and effectively deals with multicollinearity, making it particularly suited for datasets that are rich in variables yet sparse in samples. It employs Partial Least Squares Regression to efficiently categorize data, widely applied in chemometrics and bioinformatics (Berrueta et al., 2007). SVM is a powerful classifier that constructs the optimal hyperplane in high-dimensional spaces to maximize the margin between classes. With kernel tricks, it efficiently manages nonlinearities, small sample sizes, and complex, high-dimensional data, suitable for both binary and multiclass classification (Tian et al., 2014). CNN, a profound deep learning archetype, is tailor-made for image data analysis. By emulating the human visual system’s operational principles through convolutional layers, it autonomously learns hierarchical features, enabling efficient visual pattern recognition in images. The prowess of CNN in feature learning significantly enhances capabilities in image recognition, speech recognition, and natural language processing, among others. The respective strengths and limitations of the PLS-DA, SVM, and CNN models are comprehensively outlined in **Table 3**. In our approach, we structured a CNN model explicitly for processing hyperspectral data, initially preprocessing the data to meet the input requirements of the CNN model and converting sample labels into categorical format. We divided the dataset into two segments: about two-thirds designated as the training set to train the model, with the remaining third forming the test set to assess the model’s performance.

**Table 3:** The comparison of PCA, PLS-DA, SVM, and CNN models.

Algorithm	Advantages	Disadvantages
PCA	Reduces dimensionality, enhances computational efficiency, reveals hidden patterns, simplifies data visualization.	May discard useful information, sensitive to scaling, requires a separate model to make predictions.
PLS-DA	Handles multicollinearity well, suitable for small datasets, easier to interpret results.	Not suitable for non-linear problems, performance decreases with larger datasets.
SVM	Effective in high-dimensional spaces, can model non-linear relationships with appropriate kernels, robust against overfitting.	Computationally intensive, sensitive to noise and outliers, requires careful parameter tuning.
CNN	Good for large datasets, excels in feature extraction, beneficial for image data and deep learning applications.	Requires large amounts of data, high computational cost, prone to overfitting, often considered a black box.

**Figure 2:**
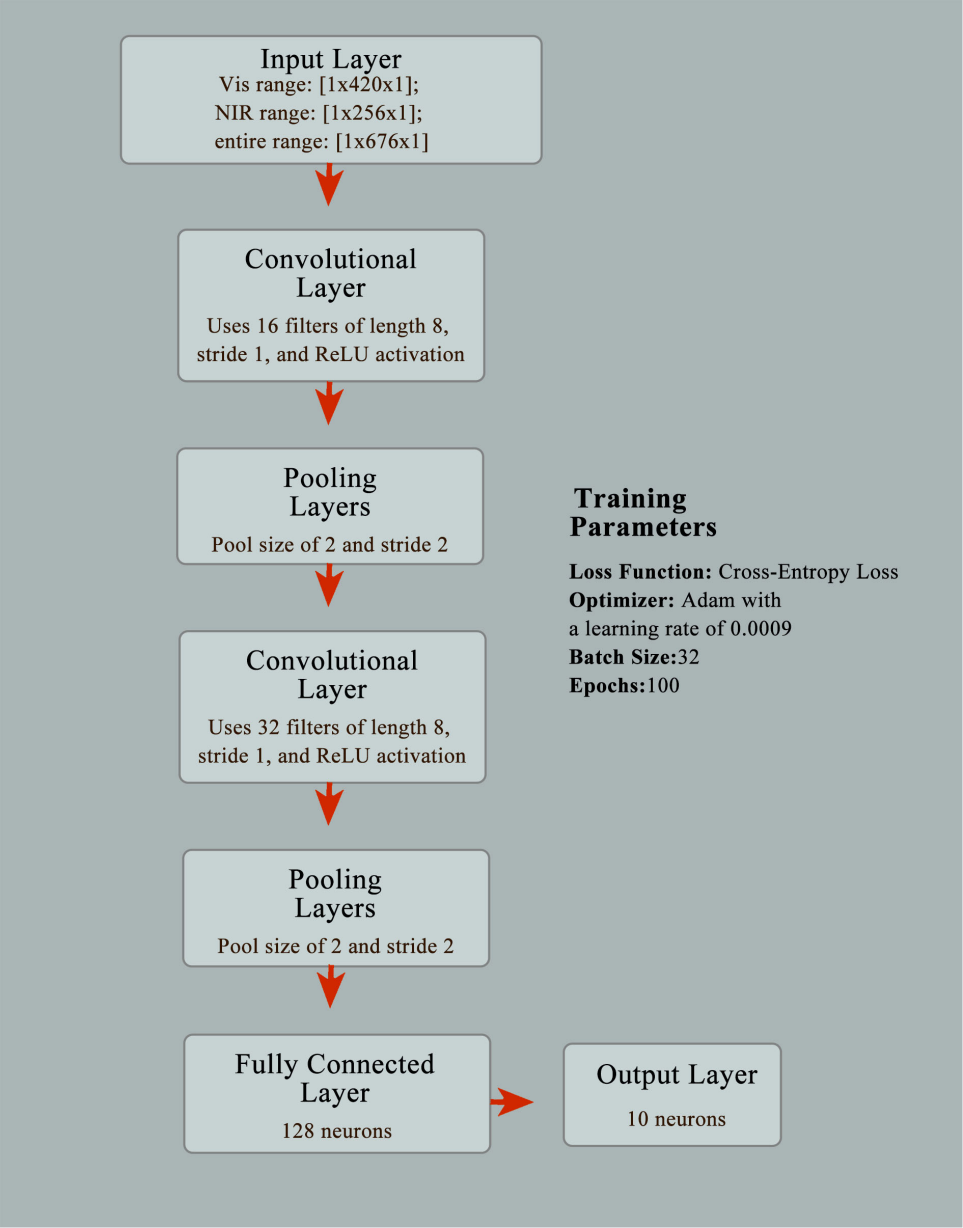
The network architecture and training parameters of CNN model.

The architecture of the CNN model was designed to include an input layer, a convolutional layer, a batch normalization layer, a ReLU activation layer, a maximum pooling layer, a fully connected layer, a softmax layer, and a classification output layer. This model aims to capture features extracted from hyperspectral data and effectively perform classification tasks. To train this network, we configured a series of training options, using the adam optimizer, setting parameters such as learning rate, maximum number of iterations, batch size, and specified shuffling of data after each round of training and a validation dataset to monitor training progress. Using the trainNetwork function, we trained the CNN model based on the aforementioned configurations and training data. The network architecture and training parameters of CNN model are depicted in **Figure 2**.

Spectral data matrices for PCA, PLS-DA, SVM, and CNN analyses were generated according to specific protocols. Each model’s data underwent initial preprocessing to minimize noise and normalize features. This step included extracting average spectra from each sample group and computing covariance matrices to evaluate relationships between various spectral features and wood species. These matrices subsequently informed the training of our models, with fine-tuning of each model’s parameters to enhance performance across diverse spectral ranges.

Prior to analysis, various preprocessing techniques were applied to the spectral data to boost model accuracy. These techniques encompassed Standard Normal Variate (SNV), Savitzky-Golay (SG) smoothing, normalization, and Multiplicative Scatter Correction (MSC). We rigorously evaluated the impact of each method to ascertain its effectiveness in enhancing the clarity and comparability of the spectral data.

In addition to accuracy, we have expanded our evaluation metrics to include precision, recall, F1-score, and the kappa coefficient. These metrics provide a more comprehensive assessment of the model’s performance across different scenarios, particularly in handling class imbalances and nuanced differentiation between species. Precision measures the accuracy of positive predictions, recall assesses how well the model captures actual positives, and the F1-score is the harmonic mean of precision and recall, offering a balance between the two in cases of uneven class distribution. The kappa coefficient, a statistical measure of inter-rater agreement for qualitative items, is adjusted for the chance agreement of categories, providing insight into the reliability of the model beyond mere accuracy.

## 3 Results

### 3.1 Spectroscopic characterization

Vis/NIR-HSI spectroscopy stands out as an exceptionally apt method for evaluating heterogeneous organic materials, including wood and wood-derived products. This advanced technique offers insights into not only the physical condition but also the chemical makeup of wood samples under examination. By scrutinizing the spectral peak positions and their configurations, it’s possible to identify the presence of specific functional groups characterized by dipole moments, enriching our understanding of the sample’s molecular structure (Sandak et al., 2020).

**Figure 3:**
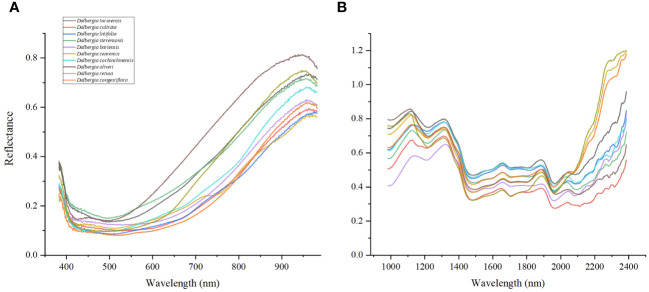
**(A)** Average Vis spectra of wood samples. **(B)** Average NIR spectra of wood samples.

**Figure 3** reveals that the ten *Dalbergia* species exhibit distinct absorption patterns, with the wood samples showing pronounced absorption peaks at wavelengths of 760, 950, 1200, 1590, 1840, 2020, and 2300 nm. The variation within the Vis spectrum can be linked to specific pigments, which show unique absorption characteristics (Palacios-Morillo et al., 2016). Notably, the peak near 950 nm is likely related to the third stretching overtone of C-H bonds, whereas the peak at 1590 nm aligns with the C-H stretching vibration in the first and second overtones (Andrés et al., 2007). The absorption at approximately 1840 nm points towards O-H stretching vibrations or the O-H-O deformation combination, often associated with moisture content (Núñez-Sánchez et al., 2016). The peak at 2020 nm is indicative of N-H stretching vibrations, generally related to the presence of lipids, carbohydrates, or protein-based organic matter (Badaró et al., 2019). Moreover, the peak around 2300 nm is presumed to be linked to fats (Krähmer et al., 2015; Lequeue et al., 2016). The identification of these specific peaks underscores the promising application of Vis/NIR-HSI spectroscopy in distinguishing between the ten *Dalbergia* species, showcasing its potential as a valuable tool for wood identification.

### 3.2 Principal component analysis

**Figure 4:**
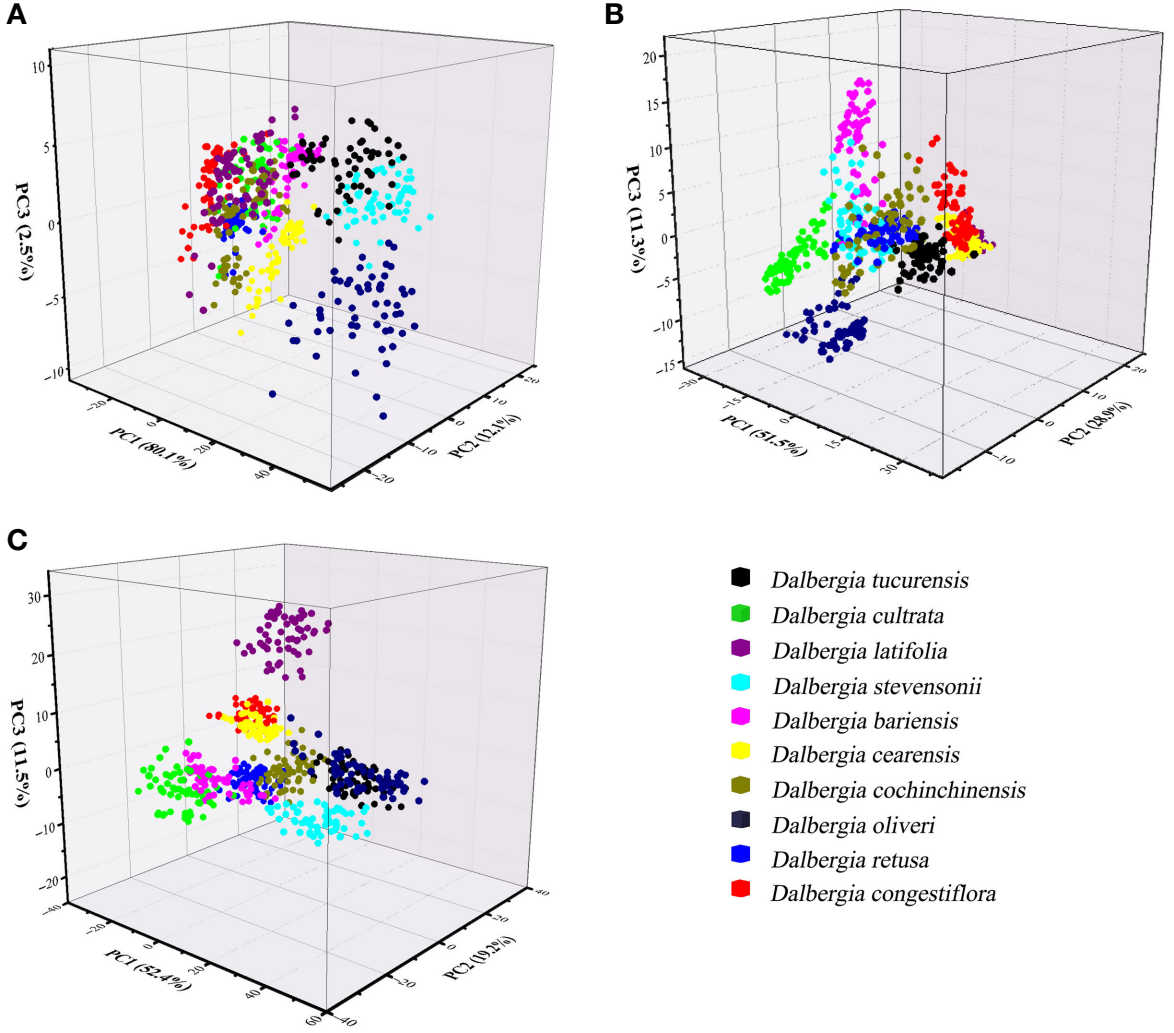
Principal Component Analysis 3D scatterplots of spectral data from distinct wood sample. **(A)** Spectral data range from 383 nm to 982 nm, **(B)** Spectral data range from 982 nm to 2386 nm, **(C)** Spectral data range from 383 nm to 2386 nm.

The dataset underwent analysis using the PCA model to explore potential clustering. PCA was conducted on wood samples across three different spectral ranges, with the initial three principal components (PCs) capturing a significant portion of the variance in these spectra. In **Figure 4A**, for instance, PC1 accounted for 80.1% of the total variance, and PC2 contributed to 12.1% of the variance. Using the NIR spectral range, the first three PCs explained 91.7% of the variance in the wood samples, with PC1 at 51.5%, PC2 at 28.9%, and PC3 at 11.3% as shown in **Figure 4B**. Additionally, **Figure 4C** demonstrates that 83.1% of the total variance was captured (PC1 = 52.4%, PC2 = 19.2%, PC3 = 11.5%), illustrating the effectiveness of PCA in highlighting the underlying structure and variance within the spectral data of these tree species.

**Figure 4** clearly illustrates that the ten *Dalbergia* species formed distinct clusters, although there was observable overlap among the wood samples of *Dalbergia bariensis* and *Dalbergia stevensonii*, as well as minor overlap between *Dalbergia tucurensis* and *Dalbergia cochinchinensis*. This indicates a certain level of spectral similarity between *Dalbergia bariensis* and *Dalbergia stevensonii*. Furthermore, the PCA results underscore the influence of spectral data range on the model’s outcomes. Among the three selected wavelength ranges, the Vis spectrum exhibited the most overlap between clusters, whereas the full wavelength range demonstrated superior clustering performance. This highlights the importance of selecting an appropriate wavelength range for wood sample identification, as an unsuitable choice may hinder the differentiation process.

**Figure 5:**
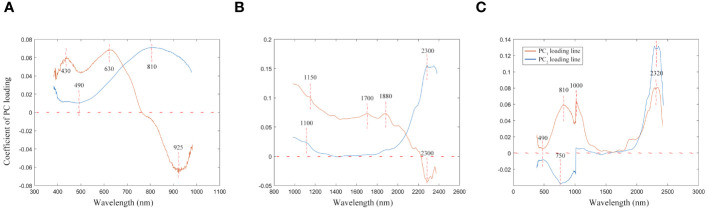
The first two PC loading lines. **(A)** Spectral data range from 383 nm to 982 nm, **(B)** Spectral data range from 982 nm to 2386 nm, **(C)** Spectral data range from 383 nm to 2386 nm.

The loading lines for the first two PC across various wavelength ranges are depicted in **Figure 5**. The figure’s curves highlight how different spectral features influence PC1 and PC2. Within the Vis wavelength range, significant contributions to PC1 are noted at 430 nm and 630 nm. These wavelengths are indicative of pigment colors and characteristics typical of mahogany-type woods, respectively. Furthermore, the PC1 loading curve exhibits a noticeable dip near 925 nm, which is associated with the absorption properties of water and other compounds in wood. For PC2, a prominent contribution is observed at 810 nm, aligning with water’s first over-absorption peak. Additionally, a dip in the PC2 curve around 490 nm captures the essence of wood’s color and surface characteristics, providing insightful details on the spectral influence of various components within the wood.

Utilizing the NIR spectrum, the wavelengths of 1150 nm, 1700 nm, and 1880 nm emerge as significant contributors to the PC1, correlating closely with the presence of moisture, lignin, and cellulose in wood, respectively. These elements play a crucial role in determining the wood’s structural integrity and chemical characteristics. Moreover, the PC1 curve exhibits a pronounced valley near 2300 nm, while the PC2 curve features a distinct peak at the same wavelength, indicating the detection of complex organic compounds within the wood, such as fatty acids and proteins. This absorption at 2300 nm is attributed to the collective and stretching vibrations of lignin, cellulose, and hemicellulose, highlighting the NIR spectroscopy’s ability to uncover detailed insights into the wood’s molecular composition and the interactions between its organic components.

When PCA analysis incorporates the full wavelength spectrum, the behavior of the PC1 and PC2 curves diverges from the patterns observed in analyses restricted to either Vis or NIR individually. As illustrated in **Figure 5C**, the wavelength of 2320 nm stands out for its substantial influence on both the PC1 and PC2 curves, signaling its significance in the dataset. Additionally, the wavelengths of 810 nm and 1000 nm make notable contributions to PC1, primarily reflecting the water content within the wood. Furthermore, the wavelength of 490 nm deserves special mention; this region is largely associated with the wood’s pigments.

**Figure 6:**
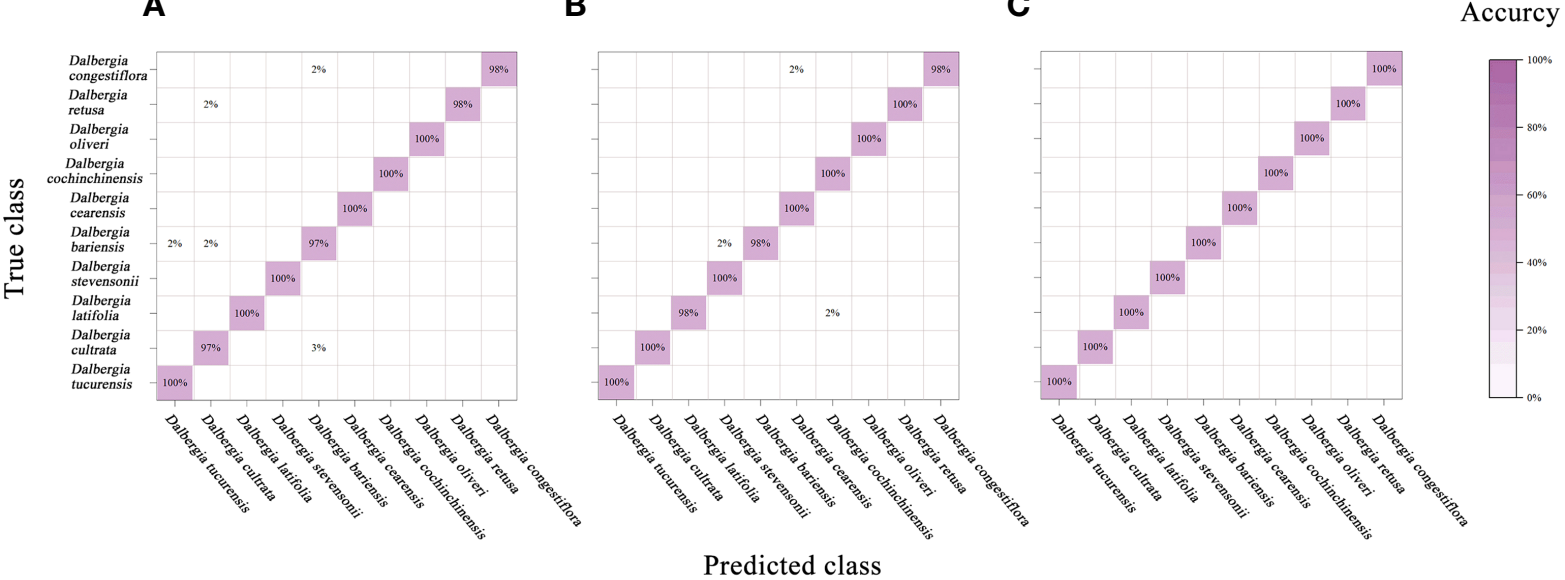
Mixing matrix of calibration set between true class and predicted class with PCA model (without preprocessing). **(A)** Spectral data range from 383 nm to 982 nm, **(B)** Spectral data range from 982nm to 2386 nm, **(C)** Spectral data range from 383 nm to 2386 nm.

In this study, we have extended the application of PCA by integrating it with a classifier, aimed at enhancing the interpretability of spectral data across various *Dalbergia* species. **Figure 6** illustrates a mixing matrix that evaluates the classification accuracy without preprocessing across three spectral ranges, underscoring how this integration helps in better understanding the spectral distinctions.

To provide a more comprehensive assessment of the model’s performance, we expanded our evaluation framework to include precision, recall, F1-score, and kappa coefficient. These metrics are crucial for addressing the spectral overlap observed among species such as *Dalbergia bariensis* and *Dalbergia stevensonii*. The results, depicted in **Table 4** and **Supplementary Table S1**, reveal that while the PCA model achieved high accuracy across the full wavelength range—91.5% in calibration and 92.5% in cross-validation—the kappa coefficient indicated lower than expected agreement. This variance in metrics like precision, recall, and F1-scores underlines the challenges in spectral classification.

**Table 4:** The accuracy of PCA model with different preprocessing method.

			383 ~982 nm			982 ~2386 nm			383 ~2386 nm	
		Calibration Set (%)	Cross-Validation Set (%)	Validation Set (%)	Calibration Set (%)	Cross-Validation Set (%)	Validation Set (%)	Calibration Set (%)	Cross-Validation Set (%)	Validation Set (%)
Preprocessing	Raw	83.5	83	84	79.5	80	80	91.5	92.5	91.5
	SNV	75.5	75	76	75	74	75.5	88	88.5	88
	SG smoothing	81	80.5	80.5	79.5	79	80.5	91	91	91.5
	Normalize	84.5	85	85	82.5	82	81	89.5	89.5	89
	MSC	83.5	83.5	84	71	71	71.5	87	87	86.5

The analysis further revealed that preprocessing methods such as SNV and SG smoothing did not significantly enhance the model’s performance within narrower spectral ranges and occasionally even led to a decrease in accuracy. This suggests potential overfitting or the loss of critical spectral information that PCA could otherwise utilize effectively. However, the comprehensive spectral range of 383 nm to 2386 nm, analyzed without preprocessing, allowed the PCA model integrated with a classifier to achieve higher accuracies—91.5% in both the calibration and validation sets, highlighting the effectiveness of PCA in capturing and utilizing the full spectrum of available features, especially when not constrained by preprocessing.

Notably, the classifier’s performance was modest across narrower spectral ranges (Vis and NIR), but showed marked improvement in the full spectral range from 383 nm to 2386 nm. This robust performance in the full wavelength range, as detailed in **Table 4**, underscores PCA’s potential in extracting meaningful information from complex spectral data, making it a viable option for scenarios requiring detailed and extensive spectral analysis. These findings suggest that while PCA can be a powerful tool for spectral classification, its efficacy is heavily dependent on the selected spectral range and the preprocessing techniques employed.

### 3.3 Results using PLS-DA

In this research, the classification of spectral data was performed using PLS-DA technique. This chemometric method, which is considered supervised technique, necessitate comprehensive understanding of the classification of each wood sample. The goal of this algorithm is to accurately assign an unknown sample to a specific class based on its spectral pattern (Berrueta et al., 2007).

**Figure 7:**
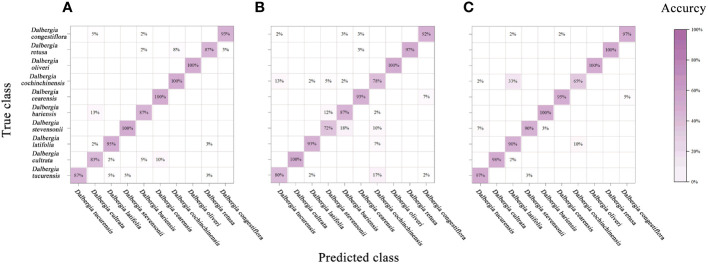
Mixing matrix of calibration set between true class and predicted class with PLS-DA model (without preprocessing). **(A)** Spectral data range from 383 nm to 982 nm, **(B)** Spectral data range from 982nm to 2386 nm, **(C)** Spectral data range from 383 nm to 2386 nm.

**Figure 7** and **Table 5** summarize the accuracy achieved by the PLS-DA model and assess the impact of various preprocessing algorithms. PLS-DA, a widely recognized classification model, excels in computational efficiency and is designed to identify the optimal functional correlation within a dataset by minimizing the sum of squared errors (Lestander et al., 2008). Despite its broad application in multivariate data analysis, the PLS-DA model displayed limited success in accurately identifying ten *Dalbergia* species, with the most promising results emerging from analyses that utilized the full spectral range for both calibration and validation sets.

**Table 5:** The accuracy of PLS-DA model with different preprocessing method.

		383 ~982 nm			982 ~2386 nm			383 ~2386 nm		
		Calibration Set (%)	Cross-Validation Set (%)	Validation Set (%)	Calibration Set (%)	Cross-Validation Set (%)	Validation Set (%)	Calibration Set (%)	Cross- Validation Set (%)	Validation Set (%)
Preprocessing	Raw	93.2	93	94	89.2	87.5	88	93.2	93.5	94
	SNV	88	87.5	88.5	88.8	86	87	88.8	87	88.5
	SG smoothing	95.8	95	96	92	89	90.5	96.5	96	96
	Normalize	95.8	95	96	90.3	87.5	89.5	95.8	95	96
	MSC	85.5	84.5	87	88	86	87	87.5	85.5	88

In our study, to diminish the adverse effects of noise, an array of preprocessing techniques was applied to the spectral data. The analysis revealed that preprocessing notably enhances the model’s performance over the raw spectral data. According to **Table 5**, integrating PLS-DA with SG smoothing yielded the highest prediction accuracy. In contrast, the SNV and MSC treatments produced somewhat inferior outcomes. Additionally, the efficacy of these methods varied across the three examined spectral ranges. The premier performance was achieved by a PLS-DA model that utilized the entire spectral range combined with SG smoothing, achieving calibration and validation accuracies of 96.5% and 96%, respectively.

An analysis of the mixing matrix uncovered significant discrepancies in the model’s ability to differentiate among species across the spectral ranges. Specifically, the NIR range’s accuracy in identifying *Dalbergia bariensis* and *Dalbergia stevensonii* was notably low, coupled with recurrent misclassifications between *Dalbergia tucurensis* and *Dalbergia cochinchinensis*. These issues were mitigated when applying the full spectral range, which also improved the kappa coefficient, underscoring its importance in reflecting the model’s overall reliability and not just accuracy. This underscores the importance of selecting an apt spectral range to ensure accurate species identification and balanced evaluation metrics (**Supplementary Table S1**).

### 3.4 Results using SVM

SVM, a supervised machine learning technique, is effective in addressing regression and classification challenges. It is a non-linear classification approach that builds a collection of hyperplanes in an infinite or high-dimensional space. The hyperplane that exhibits the maximum separation with the nearest training data point from any class ensures accurate classification (Tian et al., 2014). Unlike the PLS-DA model, SVM is not affected by the distribution of distinct sample classes.

**Figure 8:**
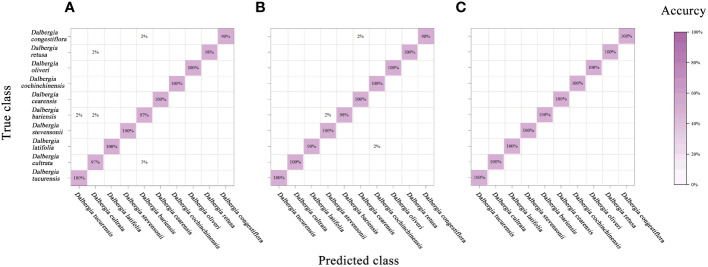
Mixing matrix of calibration set between true class and predicted class with SVM model (without preprocessing). **(A)** Spectral data range from 383 nm to 982 nm, **(B)** Spectral data range from 982nm to 2386 nm, **(C)** Spectral data range from 383 nm to 2386 nm.

Relative to the performance of the PLS-DA model, the SVM showcased enhanced effectiveness, as evidenced in **Figure 8** and **Table 6**. Notably, leveraging data across the full spectral range in conjunction with the SVM model led to improved accuracy. In parallel with the PLS-DA model, the SVM model reached peak accuracy with the application of SG smoothing. Employing SG smoothing allowed for the achievement of impeccable 100% accuracy within both the Vis and NIR spectral ranges. Furthermore, when integrating both Vis and NIR spectral ranges, the developed SVM model is capable of reaching 100% accuracy, both in the absence of data preprocessing and when employing SG smoothing, normalization, and MSC as preprocessing techniques. The only scenario where accuracy diminishes is when the SNV algorithm is applied for data preprocessing.

**Table 6:** The accuracy of SVM model with different preprocessing method.

			383 ~982 nm			982 ~2386 nm			383 ~2386 nm	
		Calibration Set (%)	Cross-Validation Set (%)	Validation Set (%)	Calibration Set (%)	Cross-Validation Set (%)	Validation Set (%)	Calibration Set (%)	Cross-Validation Set (%)	Validation Set (%)
Preprocessing	Raw	99	98	99	99.5	99	99.5	100	99.5	100
	SNV	96.5	96	97	96.8	96	96	96.5	95	97
	SG smoothing	100	99.5	100	100	100	100	100	100	100
	Normalize	99.8	99	100	100	99.5	100	100	98	100
	MSC	99	97.5	99	99.5	99	99.5	100	98.5	100

### 3.5 Results using CNN

In our study, we assessed the CNN model’s performance by applying the trained model to the calibration set and validation set, cross-validation set and calculating accuracy. Additionally, we used the confusion matrix to further analyze the model’s classification capabilities. This series of steps not only ensured the effectiveness of our model but also provided a clear framework for deep learning analysis of hyperspectral data.

**Figure 9:**
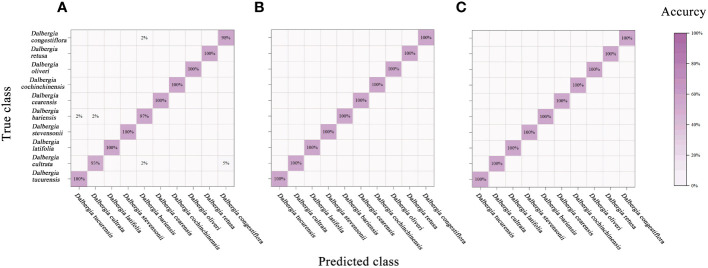
Mixing matrix of calibration set between true class and predicted class with CNN model (without preprocessing). **(A)** Spectral data range from 383 nm to 982 nm, **(B)** Spectral data range from 982nm to 2386 nm, **(C)** Spectral data range from 383 nm to 2386 nm.

**Figure 10:**
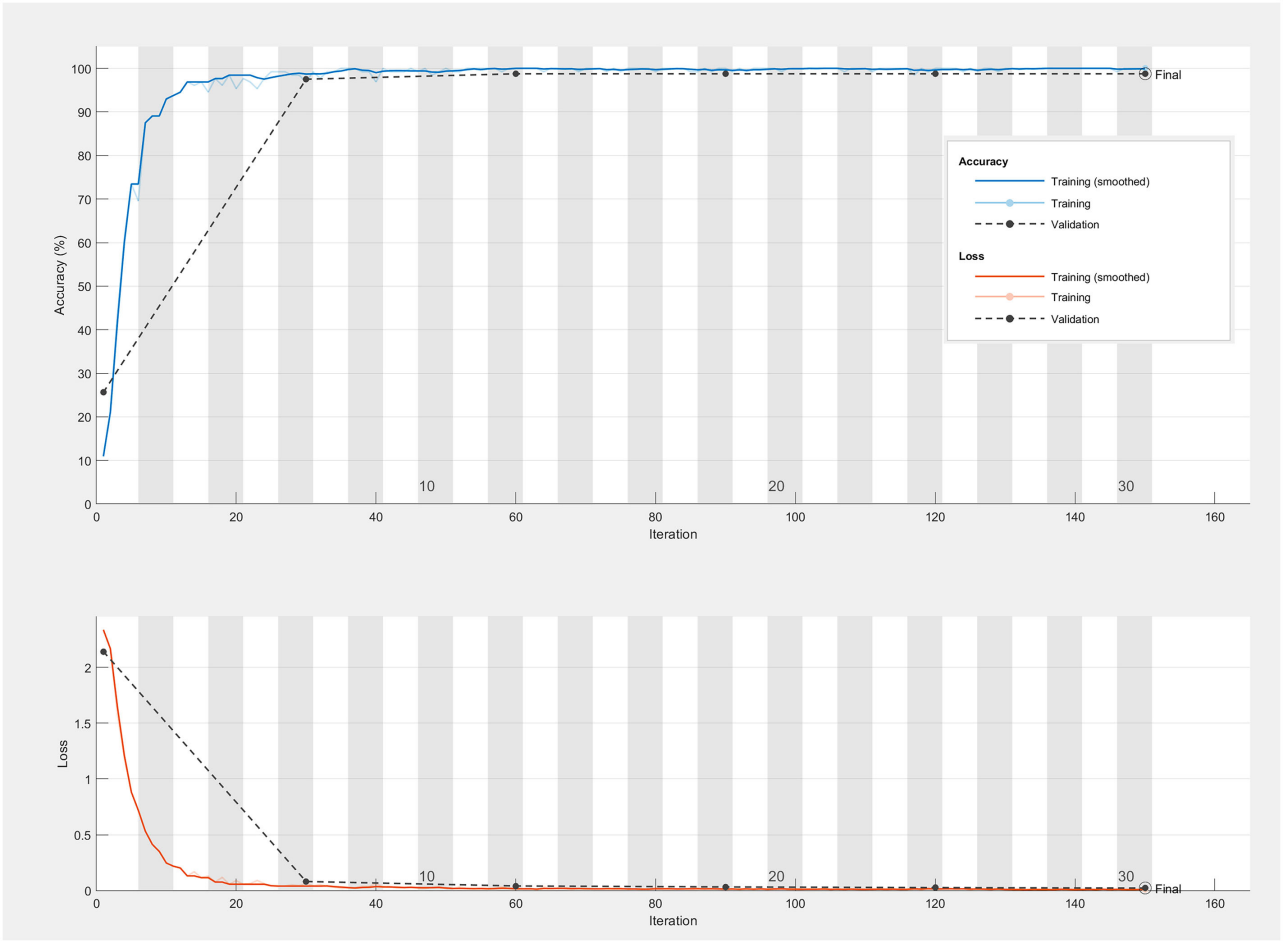
The accuracy and loss curves of CNN model range from 383 nm to 2386 nm without preprocessing.

**Figure 9** and **Table 7** encapsulate the accuracy outcomes of our CNN model’s deployment and assess the impacts of various preprocessing algorithms. **Figure 10** illustrates the accuracy and loss curves for the CNN model across the spectrum, ranging from 383 nm to 2386 nm, when no data preprocessing is applied. It’s evident that the accuracy achieved by the CNN model across different wavelength ranges substantially surpasses that of the PLS-DA model. In comparison with the SVM model, an impressive 100% accuracy is attainable for each wavelength range model upon selecting a suitable data preprocessing technique. However, it’s crucial to acknowledge that, contrary to traditional models, employing the SG smoothing algorithm in conjunction with the CNN model tends to deteriorate the model’s predictive performance. Techniques such as SNV, Normalization, and MSC demonstrate their utility across diverse spectral ranges. To conclude, the selection of an apt data preprocessing method when constructing a CNN model presents a more nuanced challenge, underscoring the importance of tailored preprocessing strategies for optimizing model performance.

**Table 7:** The accuracy of CNN model with different preprocessing method.

			383 ~982 nm			982 ~2386 nm			383 ~2386 nm	
		Calibration Set (%)	Cross-Validation Set (%)	Validation Set (%)	Calibration Set (%)	Cross-Validation Set (%)	Validation Set (%)	Calibration Set (%)	Cross- Validation Set (%)	Validation Set (%)
Preprocessing	Raw	98.8	98	99	100	98	98.5	100	99	100
	SNV	100	100	100	100	98	98	100	99.5	100
	SG smoothing	99.5	97	98.5	99	98.5	99.5	99	99	99.5
	Normalize	100	97.5	99.5	99.7	98.5	99	100	99	99.5
	MSC	100	100	100	100	99	99.5	99.3	96	97.5

To conclude, our CNN model not only excels in terms of accuracy but also maintains high levels of precision, recall, F1-scores, and kappa coefficients (**Supplementary Table S1**), illustrating its robustness and the effectiveness of our deep learning approach in handling hyperspectral data classification.

## 4 Discussion

In recent years, the Vis/NIR technique has garnered attention for identifying precious woods [41], with extensive exploration into spectroscopic methods for rosewood identification (Yang et al., 2012; Snel et al., 2018; Raobelina et al., 2023). Yet, the selection of the most appropriate wavelength range has been largely overlooked. Using an unsuitable wavelength range not only diminishes identification accuracy but also escalates equipment costs. Our study aims to address this issue by constructing a classification model leveraging spectral data within the Vis, NIR, and the entire ranges.

### 4.1 Scientific basis for wavelength selection

The strategic selection of specific wavelength ranges was meticulously informed by a thorough analysis of the unique spectral characteristics of *Dalbergia* woods. As depicted in **Figure 3**, notable differences in the Vis and NIR spectra are evident, with prominent absorption peaks at 760, 950, 1200, 1590, 1840, 2020, and 2300 nm. These peaks are indicative of molecular bond vibrations involving O–H, C–H, C–O, and N–H, which illuminate the complex chemical interactions within the wood samples. This detailed spectral data underscores the capability of Vis/NIR-HSI technology for precise identification of wood samples.

By selecting the entire wavelength range of 383 nm to 2386 nm, we captured the most extensive spectral information, thereby significantly enhancing the robustness of our predictive models. This choice highlights the importance of adopting a comprehensive spectral perspective that spans both Vis and NIR light, fully leveraging the chemical and physical properties of the samples. This integration of spectral insights optimally refines our wavelength selection strategy, tailoring it to meet the specific demands of HSI applications and ensuring that critical features crucial for differentiating wood species are meticulously captured.

This methodical selection process not only is scientifically justified but also enhances the practicality and accuracy of wood species identification using HSI technology, thereby improving both the effectiveness and precision of our analytical approach.

### 4.2 Optimal model selection

In our experiments, the integration of PCA with classifiers demonstrated considerable variability in performance across different spectral ranges. Utilizing PCA across the full spectral range yielded excellent classification accuracy, benefiting from the broad coverage that captures a diverse range of chemical interactions crucial for differentiating wood species. This suggests that the use of the full spectral range significantly enhances the effectiveness of PCA.

PLS-DA models excelled in managing high-dimensional data and addressing multicollinearity, proving particularly effective for datasets that are variable-rich but sample-sparse. Concurrently, SVM models demonstrated their efficacy in constructing optimal hyperplanes in high-dimensional spaces, significantly improving class separation, which is ideal for managing smaller samples or more complex data scenarios. Within the context of hyperspectral data for wood species analysis, CNN models provided distinct advantages over PLS-DA and SVM models. CNNs inherently learn complex spatial and spectral features from hyperspectral data without the need for manual feature extraction, an invaluable attribute given the complexity of the data. Their deep architecture facilitates effective management of high-dimensional data, enhancing classification and identification accuracy by capturing subtle variations in wood data. Moreover, CNNs exhibit robust generalization capabilities, maintaining high performance across a variety of new and diverse wood samples. Despite their requirement for substantial computational resources and large datasets, the exceptional ability of CNNs to handle hyperspectral images—especially in learning intricate features and managing high-dimensional data—establishes them as a highly promising tool for qualitative research in wood species. The CNN model developed in this study delivered reliable results across three wavelength ranges, outperforming the PLS-DA model in terms of accuracy, and equaling the reliability of the SVM model. However, this investigation was limited to spectral data of wood samples, with spatial data aspects remaining unexplored. Thus, CNN models based on hyperspectral data have a broader potential for research. Additionally, we found that CNN models tailored to different wavelength ranges require uniquely optimized data preprocessing approaches, underscoring the necessity for precise preprocessing method selection according to the spectral data types and test samples.

### 4.3 Effectiveness of preprocessing techniques

SNV, SG smoothing, normalization, and MSC are four preprocessing techniques widely used in spectroscopic data analysis to improve data quality before further analysis (Chen et al., 2011). SNV effectively corrects scatter effects and removes multiplicative interferences from spectra by normalizing each sample—subtracting the mean and dividing by the standard deviation, which significantly reduces variability among samples. SG smoothing, a robust filtering technique, applies a polynomial regression over a moving window across the data, adeptly reducing noise while preserving critical features such as peak height and width. Conversely, normalization scales the data to a specific range or unit norm, ensuring that variations in the dataset reflect true differences in the measurements, thereby facilitating comparison across different samples or datasets. MSC enhances spectral comparability by adjusting for variations in light scattering and absorption based on a reference or mean spectrum, thus improving analytical accuracy.

In comparison, SNV and MSC are primarily focused on correcting scatter effects and reducing sample variability, thereby enhancing spectral quality by compensating for physical discrepancies. SG smoothing, tailored for noise reduction, and normalization, aimed at calibrating the data scale, collectively work to ensure consistency and augment data clarity and interpretability. This study adopts a comprehensive preprocessing approach that integrates SNV, SG smoothing, normalization, and MSC to optimize the spectral data for analysis.

Our analysis assessed how these preprocessing techniques impact the performance of four models: PCA, PLS-DA, SVM, and CNN. Each model exhibits unique responses to preprocessing due to its specific data processing needs. PCA showed optimal performance without preprocessing, achieving accuracies of 91.5% in both the calibration and validation sets, and 92.5% in the cross-validation set across the full spectral range. Techniques such as SNV and SG smoothing tended to decrease PCA’s accuracy, potentially by altering essential spectral features. PLS-DA benefits from preprocessing that corrects multicollinearity and enhances data uniformity, integral to its regression-based framework. SVM gains advantages from methods like SG smoothing, which clarify class boundaries by reducing noise, crucial for its hyperplane-based classification strategy. CNN’s response varied depending on the preprocessing method; techniques that preserve the original spectral integrity while reducing noise, such as MSC, generally bolster CNN performance by enhancing the model’s capacity to discern discriminative features from complex hyperspectral images.

In summary, while certain models like PCA favor minimal preprocessing to retain detailed spectral information, others like PLS-DA and CNN may require more intensive preprocessing to effectively prepare data for analysis. This highlights the necessity of aligning preprocessing techniques with the specific requirements of each model to maximize performance in HSI applications. Future research should focus on developing preprocessing strategies that leverage the strengths of each model to improve accuracy and effectiveness.

### 4.4 Comparison with traditional methods

Our study demonstrates that HSI combined with machine learning significantly surpasses traditional wood identification methods such as microscopy or chemical assays, primarily in terms of non-destructiveness, efficiency, and accuracy. Traditional techniques, often labor-intensive and destructive, can compromise sample integrity and are limited in their ability to differentiate closely related species due to their reliance on visible morphological or chemical characteristics.

In contrast, HSI provides a comprehensive analysis by capturing a continuous spectrum for each pixel in the image, offering detailed insight into both the chemical and physical properties of wood samples across a broad spectral range. This method allows for the identification of subtle differences in wood species that traditional methods might miss. For instance, HSI can detect unique spectral signatures associated with specific chemical bonds and structures within the wood, which are indicative of different species.

Moreover, when comparing Vis/NIR HSI to conventional Vis and NIR spectroscopy techniques, HSI offers enhanced capabilities. While Vis and NIR spectroscopy provide valuable data regarding the wood’s composition, they typically do so at discrete wavelengths and often require prior knowledge of which wavelengths are most relevant for differentiation. Vis/NIR HSI, on the other hand, captures data across a contiguous spectral range, from visible to near-infrared, allowing for a more flexible and detailed analysis without the need for predefined spectral bands.

The integration of machine learning with HSI further amplifies these advantages. Machine learning algorithms can process the complex, high-dimensional data generated by HSI, efficiently classifying wood species based on learned spectral patterns. This combination not only increases the accuracy of species identification but also enhances the speed and automation of the process, making it highly suitable for large-scale and real-time applications.

These advanced capabilities of HSI and machine learning contribute significantly to conservation efforts and sustainable forestry practices by providing a rapid, accurate, and non-destructive means of identifying wood species, essential for combating illegal logging and promoting biodiversity preservation. As such, HSI represents a substantial improvement over both traditional methods and conventional Vis/NIR spectroscopy, promising a new standard for the field of wood identification.

### 4.5 Future directions in wood identification technology

Future research in wood identification leveraging HSI and machine learning should prioritize the integration of advanced algorithms such as Generative Adversarial Networks (GANs) and deep reinforcement learning to enhance both accuracy and efficiency. By integrating HSI with data from microscopic structures, chemical compositions, and geographic tagging, system robustness can be significantly improved through multimodal analysis. Expanding the hyperspectral database to encompass a broader range of species and utilizing adaptive learning and transfer learning techniques will facilitate adjustments to species-specific characteristics and environmental variations, thereby advancing the limits of accuracy and processing speeds.

In practical applications, specific models such as PCA, PLS-DA, SVM, and CNN each follow unique optimization pathways. PCA is essential for reducing dimensionality while preserving critical spectral data. PLS-DA can be further refined to more effectively manage datasets that are rich in variables. SVM can be optimized through kernel functions to enhance robust classification capabilities, and CNN can be tailored through network architectures to improve deep feature learning. These advancements are crucial for applications in ecological conservation, biodiversity preservation, and timber regulation, providing precise identification capabilities that aid in combating illegal logging and supporting sustainable forestry practices.

## 5 Conclusions

Our study marks significant advancements in the non-destructive identification of *Dalbergia* species through the use of Vis/NIR HSI combined with sophisticated modeling techniques such as PCA, PLS-DA, SVM, and CNN. The implementation of these methods greatly enhances both the accuracy and efficiency of wood species identification, offering essential support for conservation efforts and the enforcement of trade regulations. Notably, with optimal preprocessing, both SVM and CNN models achieve 100% accuracy across NIR and full spectral ranges.

Furthermore, our results highlight the superiority of Vis/NIR HSI coupled with machine learning over traditional wood identification methods such as microscopy or chemical assays. By utilizing continuous spectral data and advanced computational models, HSI provides a more comprehensive and non-destructive analysis, which is highly efficient and accurate. This capability is especially critical for distinguishing closely related species and supporting sustainable forestry practices. Additionally, compared to conventional Vis and NIR spectroscopy, Vis/NIR HSI offers more detailed insights due to its ability to capture a continuous spectral range, thus enabling a more flexible and comprehensive analysis without the limitations of predefined spectral bands.

Looking ahead, incorporating more sophisticated machine learning algorithms, such as GANs and deep reinforcement learning, shows great promise for further improving identification accuracy. Future research should also aim to expand the hyperspectral database to encompass a wider range of wood species and integrate data from various sources, such as microscopic structure images and chemical composition analysis, to enhance the system’s robustness and generalization capabilities.

These technological advancements not only offer immense potential for applications in ecological conservation and biodiversity preservation but also play a crucial role in regulating the timber industry. By providing a precise, rapid, and non-destructive method of identification, these technologies ensure that only legally sourced timber is traded, thereby playing a vital role in combating illegal logging and preserving global biodiversity.

## Data availability statement

The raw data supporting the conclusions of this article will be made available by the authors, without undue reservation.

## Author contributions

ZC: Writing – original draft, Writing – review & editing. XX: Funding acquisition, Writing – review & editing. HW: Software, Writing – review & editing. HG: Methodology, Writing – review & editing. GW: Supervision, Writing – review & editing. GN: Investigation, Writing – review & editing. TC: Investigation, Writing – review & editing.

## Supplementary material

Supplementary Table 1 Model parameter.
